# Evaluation of cholera surveillance system in Osu Klottey District, Accra, Ghana (2011-2013)

**DOI:** 10.11604/pamj.2017.28.224.10737

**Published:** 2017-11-13

**Authors:** Eric Yirenkyi Adjei, Keziah Laurencia Malm, Kofi Nyarko Mensah, Samuel Oko Sackey, Donne Ameme, Ernest Kenu, Marijanatu Abdulai, Richael Mills, Edwin Afari

**Affiliations:** 1 Ghana Field Epidemiology and Laboratory Training Program (GFELTP), Accra, Ghana; 2 Ghana Health Services, Accra, Ghana; 3 Korle Bu Teaching Hospital, Accra, Ghana

**Keywords:** Predictive value positive, surveillance, case fatality rate, evaluation, Osu Klottey, stakeholders, indicators

## Abstract

**Introduction:**

Cholera is an acute illness characterized by profuse watery diarrhea. It is caused by vibrio cholera subgroup 01 and 0139. Rapid administration of fluid replacement therapy and supportive treatment can reduce mortality to around 1%. By the close of 2011, 10,628 cases and 100 deaths were reported in Ghana with a case fatality rate of 0.99. It is important to evaluate the cholera surveillance system in Ghana to determine if it is meeting its objective.

**Methods:**

The study was conducted in Osu Klottey district in the Accra Metropolitan area in January 2014. We assessed the operations (attributes and performance) of the surveillance system for cholera using CDC guidelines (2001). Surveillance data records at the district level from 2011-2013 were extracted and analyzed for frequency using Microsoft excel. Stakeholders and key informants were interviewed using structured questionnaire. Records were also reviewed at some health facilities and at district levels.

**Results:**

In 2011 and 2012, case fatality rates (1.3% and 0.65%) respectively. Males were mostly affected. The most affected age group was 20-29. In 2011, Predictive value positive was 69.2% and 50% in 2012.Cholera peaked in March 2011 and April 2012. The Government of Ghana funded the system. The system is sensitive, simple, stable, flexible, acceptable and representative. It was also useful and data quality was relatively good. Predictive Value Positive was also good.

**Conclusion:**

The surveillance system is achieving its set out objectives. The system is sensitive, simple, stable, flexible, and acceptable. Predictive value positive was good.

## Introduction

Cholera is an acute illness characterized by profuse watery diarrhea. It is caused by vibrio cholera subgroup 01 and 0139 [1]. Vibrio cholera syndrome range from asymptomatic infections to cholera gravis. WHO estimate that 90% of episodes of cholera are of mild to moderate severity and are difficult to distinguish clinically from other causes of acute diarrhea. Cholera can be rapidly fatal in severe cases, and if left untreated, can result in up to 50% mortality. Rapid administration of fluid replacement therapy and supportive treatment can reduce mortality to around 1% [2]. Cholera caused by Vibrio cholera continues to be a global threat to public health and a key indicator of lack of social development. The number of cholera cases reported to WHO during 2006 rose dramatically, reaching the level of the late 1990s. In 2006, 236 896 cases were notified from 52 countries, including 6311 deaths, an overall increase of 79% compared with the number of cases reported in 2005 [1]. Once common throughout the world, the infection is now largely confined to developing countries in the tropics and subtropics. Developing countries are mostly affected because of their lack of resources, infrastructure and disaster preparedness systems. It is prevalent in Africa and portions of the Middle East, Asia, and South and Central America [3]. In Nigeria, a surveillance system was set up to evaluate cholera and other disease surveillance system, to detect disease early and monitor, so that the best available evidence would be used for decision making. The state government funds the system. A highly sensitive case definition was used to capture any patient aged 5 years or more who develops acute watery diarrhea with vomiting or no vomiting. Case definition however was simple, surveillance was complex due to laboratory confirmation. A passive surveillance system which becomes active during outbreak has official and casual sources of information. The period between outbreak commencements, verification and reaction was 24 - 48 hrs [4]. In 2013, the overall case fatality rate reported globally was 1.63%, yet, 17 countries reported case fatalities between 1% and 5% and 4 countries reported case fatalities less than 1%. If timely and proper treatment is given, case fatality rate should remain below 1% [5]. Cholera was widespread during the seventh pandemic in 1961. It began in Indonesia and in West Africa, Ghana was affected. In Ghana, public health officials are still battling with it, as in 2011 alone, 10628 cases were recorded with about 0.9% death with a case fatality rate of 0.99 [6]. Cholera surveillance system is passive. During outbreaks, it becomes active. The surveillance system in Ghana has an objective to ensure that cases are not missed, and if detected, would be managed right and on time. Stool specimen would also be transported in Cary-Blair medium to laboratory for confirmation. Cholera surveillance system is obliged to be evaluated to find out if it is meeting its objectives. This is because cholera is endemic in Ghana and as soon as it occur, public health action must be swift. Objective of this paper is to assess whether the surveillance system is effective, and also to assess attributes of the system.

## Methods

This surveillance evaluation was carried out in the Osu Klottey district in the Greater Accra Region of Ghana. It is one of the districts of Accra Metropolis and has a population of 141,127. We assessed the operations (attributes and performance) of the surveillance system for cholera in the district in January 2014 using CDC guidelines (2001). This was done by scientifically reviewing and analyzing surveillance data records from 2011-2013 at the district. This information was obtained from the disease control unit of district health directorate. Interviews checklist of stakeholders and key informants was used based on the CDC guidelines on surveillance evaluation system to obtain evidence from informants and other stakeholders at all levels. These interviews were done a using structured questionnaire. Data registers were also reviewed at some health facilities and at the district, regional, and national levels. Frequencies were obtained from data extracted from the data records with Microsoft Excel. Ethical approval was sought from the director of Accra Metropolitan Health Directorate.

## Results

**Figure 1:**
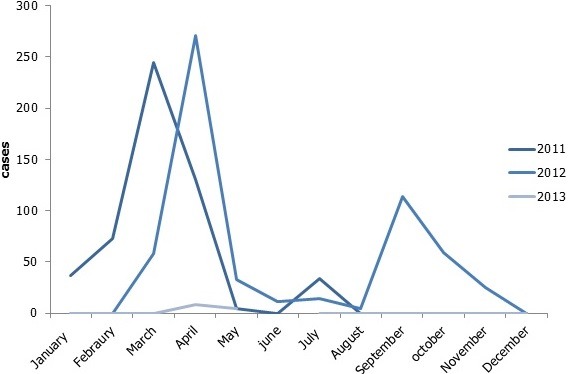
Trend of cholera cases in Osu Klottey from 2011-2013

Males were mostly affected by cholera in Osu Klottey from 2011-2013. The most affected age group was 20-29 years (Table 1). Out of the 1056 cases, there were 10 deaths giving overall case fatality of 0.95%. For 2011 and 2012, 546 and 496 cases were recorded respectively. There were 7 deaths in 2011 and 3 deaths in 2012 respectively. Case fatality rates (1.3% and 0.65%) respectively. In 2011, cases started in January and peaked in March. It decreases and came to zero in June. Cases then increased again in July and started decreasing in May till December. In 2012, cases started in March, peaked in April and decreases until August. It peaked again in September and started decreasing in October until December. In 2013, cases started in April and ended in May (Figure 1). In 2011, out of 13 cases taken to the laboratory, 9 were positive. Predictive value positive was 69.2. In 2012, out of 24 samples taken to the laboratory, 12 were positive. In 2013, there were no positives (Table 2).

**Table 1:** Background characteristics and frequency of cholera cases, Osu Klottey, 2011-2013

Characteristics	Frequency
**Sex**	1056
Male	667
Female	389
**Age group (years)**	
0-9	31
10-19	125
20-29	465
30-39	198
40-49	117
50-59	59
60-69	36
70+	25

**Table 2:** Predictive value positives for cholera surveillance in Osu Klottey, 2011-2013

	Year		
	2011	2012	2013
**Total cases tested**	13	24	2
**Total cases positives**	9	12	0
**PVP (%)**	69.2	50	0

### Operations of the surveillance system

**Data collection, analysis and use of data:** The case base forms had sufficient information to capture the necessary data for use. Also patients were followed up by community based volunteers and disease control officers for information about risk factors and there was patient's privacy and data confidentiality because cases sent did not include names. Results were being used in policy-making decisions but analysis was not sufficient and complete because the only analysis was an epidemic curve drawn at the regional level. Each level and shareholder receives information it needed and data was shared in a timely fashion either weekly or monthly. There was also feedback from all participants. The systems structure from community, facility, district, region, national and international encouraged feedback from all participants.

**Resources used to operate the surveillance system:** The Government of Ghana (GOG) and other partners (WHO, CDC, UNICEF) were often the funding sources for the cholera surveillance system. The system was integrated with other diseases under surveillance. Hence resources from other diseases also benefited the system. The personnel were Ghana Health Service employees and equipment used was partly provided by GOG and other partners from other disease programs. As such the cost of operation of the system was quite difficult to determine.

**Figure 2:**
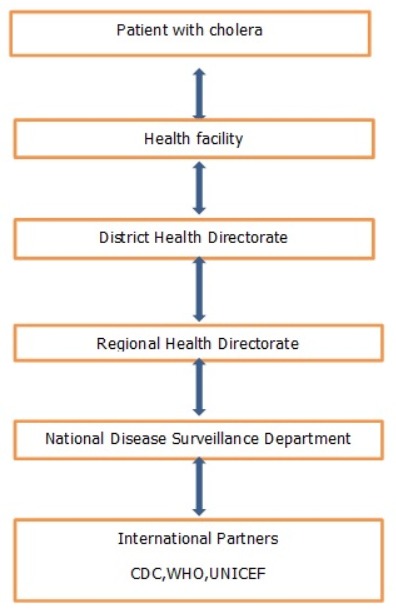
Flow diagram for reporting cholera cases from patient through to international partners in Osu Klottey district

**Flow chart:** Cholera patients reported to the health facilities through their care takers. The medical attendant present at the facility provided the diagnosis and registered the case in a consulting room register. The information Officer at the facility goes through the register collects and collates the number of cases and deaths to be reported to the district health directorate and subsequently to the regional and national level (Figure 2). Health facilities reported daily every cholera case by hard copy to the district and every Monday on weekly bases as well as on monthly bases. Submission by the district to region was done on every Monday or Tuesday of the week but on every 5th day of the following month by hard copy or by text. Submission by region to national was by electronic mail but sometimes by hard copy. During outbreaks submission of report was done daily. The national Division of Health Systems and Services Development (DSD) shares the data with the international partners. Moreover, in all this processes feedback was given at the various levels. Feedback from national was by epidemiological bulletin, or verbal. Feedback from the region was by electronic mail. Feedback from district was also verbal.

**System's attributes:** The system is very simple and acceptable. It is representative, flexible and useful. Data quality is good as well as timeliness. Predictive value positive is good and the system is sensitive. The system is simple as the information required to establish a case is easy as seen from the case definition. Also case base forms are easy and simple to understand. Levels of reporting are few. Acceptability reflects the willingness of persons and organizations to participate in the surveillance system. The system was representative because the occurrence of cholera over months in Osu Klottey and its distribution in the population by place and person was described .The system was found to be flexible because it adapt to changing information needs or operating conditions with little additional time, personnel or allocated funds. The system detects disease or protective exposure of public importance; timely estimates of the magnitude of morbidity and mortality. It also detect trend, hence it was useful. The amount of time between the onset of disease and the time it was reported to health facilities is between 12-24 hours. Hence timeliness was good. The system is stable due to its integration with other diseases with greater funding sources. The system is sensitive because it was able to detect cholera cases. Out of 39 cases sent to the laboratory, 21 were positive thus, the Predictive Value Positive (PVP) was 53.8%

**Objectives and usefulness of the surveillance system**: the surveillance system has achieved its set out objectives. The surveillance system detected and responded promptly to cases of watery diarrhea. This was done by health workers and community base volunteers. All 39 samples taken to the laboratory were in Cary Blair. Between 2011 and 2013, 1056 suspected cases were detected.

## Discussion

**Statement of principal findings**: in 2011 546 cases were reported with 7 deaths (CFR = 1.3%). In 2012 496 cases were reported with 3 deaths (CFR = 0.6%). In 2013, (CFR = 0.0). On the average (CFR = 0.95). The case fatality rates of the surveillance system seen in this study in 2011(1.3%) and 2012(0.6%) is lower than what was seen in Nigeria by Boshorun and friends, (4.1% in 2011 and 1.29 in 2012 by week 26). The positive predicted value of the surveillance system seen in this study (58.3%) is higher than what was seen in Nigeria by Bashorun and friends, 33% [4]. Acceptability of the system is high among all stakeholders interviewed. The 20-29 year group, the most economically active, are mostly affected. So measures such as education on cholera must be given to this age group to avoid low productivity in the future.

**Strength and weaknesses of the study**: Cholera surveillance system meets its objectives because of clear field guidelines and well trained staff. Surveillance system was set up for early detection and monitoring towards evidence-based decision. There was availability of reliable data, few of the data did not have names and sexes with them.

**Strength and weaknesses in relation to other study**: The system is passive and active during outbreaks. Sources of information is formal and informal. Government of Ghana funds system. This concurs with the study by Bushorun and friends. This study was effective in influencing actions and preventions, the size of my data was small as compared to the work by the Nigerians. Standardized laboratory with consistent supply of Cary Blair was used for case confirmation contrary to the work by the Nigerians where samples was taken sometimes, without the transport media.

**Discussion of important differences in results**: Case fatality rate in this study was lower than what was seen in the study in Nigeria by Bushorun and friends [5]. This means that cholera cases in Osu klottey were treated properly and timely, with respect to average (CFR = 0.95) 2011-2013. However, timely and proper treatment is not given to cholera cases in Nigeria [5]. This may be due to the fact patients do not report to health centers early to be attended to. Higher Positive Predictive value (58.3%) in my study as compared to the study by the Nigerians (33%) may be due to the higher number of cases sent to the laboratory by the Nigerians. It may also be due to the sensitivity of case definition used.

**Meaning of the study**: The ministry of health must educate, cholera transmission and prevention must be targeted to the youth especially 20-29 year group so that morbidity will be reduced in that age group. Disease control officers must be trained on data management to improve data quality.

**Unanswered questions and future research**: Do stakeholders come together occasionally to discuss areas where research must be strengthened based on surveillance reports? This would direct policy implementation. Frequency of collaboration of disease surveillance reports by stakeholders must be researched unto.

## Conclusion

The surveillance system is meeting its objective because it detects and responds to outbreaks early. The system is sensitive, simple, stable, flexible, acceptable and representative. It was also useful and data quality was relatively good. Predictive Value Positive was also good.

### What is known about this topic

- Ghana has a cholera surveillance system at the district, regional and national levels; this system is only active during outbreaks.

### What this study adds

- The cholera surveillance system in Osu Klottey district has a good Predictive Value Positive and is achieving its set out objectives.

## Competing interests

The authors declare no competing interest.
